# Three Months of Text4Hope-Addiction Support Program mitigates substance craving and improves mental health.

**DOI:** 10.1192/j.eurpsy.2023.397

**Published:** 2023-07-19

**Authors:** G. Obuobi-Donkor, R. Shalaby, W. Vuong, B. Agyapong, A. Gusnowski, S. Surood, V. I. O. Agyapong

**Affiliations:** ^1^Psychiatry, Dalhousie University, Halifax; ^2^Psychiatry, University of Alberta; ^3^Psychiatry, Alberta Health Services, Edmonton, Canada

## Abstract

**Introduction:**

Problematic substance use is rising, and other mental health conditions like anxiety and depression correlate with substance abuse. Diverse interventions to reduce this effect are emerging. Supportive text messages offer the prospect of improving symptoms of drug misuse and other associated comorbidities.

**Objectives:**

The study aims to evaluate the impact of the Text4Hope-Addiction program in mitigating craving, anxiety, and depression symptoms in subscribers.

**Methods:**

Individuals self-subscribe to Text4Hope Addiction program by texting “Open2Change” to 393939 to receive daily addiction-related text messages for three months. Subscribers are invited via text message to complete online questionnaires which assess cravings, anxiety, and depressive symptoms using the Brief Substance Craving Scale, Generalized Anxiety Disorder-7 Scale, and Patient Health Questionnaire-9 on subscription (baseline), six weeks and three months. Data were analyzed using SPSS version 25 with descriptive and inferential statistics. Satisfaction responses were used to assess various aspects of the Text4Hope- Addiction program.

**Results:**

There was a significant difference in the mean baseline and three-month BSCS scores ( -2.17, 95% CI of -0.62 to -3.72), PHQ-9 scores (-5.08, 95% CI of -1.65 to -8.51), and the GAD-7 scores (-2.93, 95% CI of -0.48 to -5.56). Participants agreed that the supportive text messages helped them cope with addiction-related stress (89%), anxiety (81%) and depression (69%).

**Conclusions:**

The Text4Hope-Addiction program effectively reduced cravings, anxiety, and depression among subscribers, with high satisfaction rates for the program. Healthcare practitioners and policymakers should consider implementing supportive text-based strategies to complement conventional treatments for addiction.

**Disclosure of Interest:**

None Declared

